# The influence of cognitive load and vision variability on postural balance in adolescents with intellectual disabilities

**DOI:** 10.3389/fneur.2024.1385286

**Published:** 2024-05-31

**Authors:** Ghada Jouira, Dan Iulian Alexe, Cristina Elena Moraru, Ghazi Rekik, Cristina Ioana Alexe, Marius Alin Marinău, Sonia Sahli

**Affiliations:** ^1^Research Laboratory Education, Motricité, Sport et Santé (EM2S) LR19JS01, High Institute of Sport and Physical Education of Sfax, University of Sfax, Sfax, Tunisia; ^2^Department of Physical and Occupational Therapy, “Vasile Alecsandri” University of Bacău, Bacău, Romania; ^3^Department of Physical Education and Sport, “Alexandru Ioan Cuza” University of Iaşi, Iaşi, Romania; ^4^Tanyu Research Laboratory, Taipei, Taiwan; ^5^Department of Physical Education and Sports Performance, “Vasile Alecsandri” University of Bacău, Bacău, Romania; ^6^Department of Physical Education, Sport and Kinetotherapy, University of Oradea, Oradea, Romania

**Keywords:** intellectual disabilities, adolescent, postural balance, cognitive load, vision

## Abstract

**Introduction:**

This study aimed to investigate the impact of cognitive load, particularly its escalation during the execution of the same test, under varying vision conditions, on postural balance among adolescents with intellectual disability (ID).

**Methods:**

Twenty adolescents underwent balance assessments under different visual conditions (Open Eyes (OE), Closed Eyes (CE), Flash, Goggles, Visual Stimulation (VS)) and task settings (Single Task (ST), Dual Task (DT) without challenges, and DT with challenges). The cognitive test was assessed using Verbal Fluency (VF).

**Results and discussion:**

Significant effects were found for Task (*p* < 0.001, ηp^2^ = 0.85), indicating that CoP values significantly increased (*p* < 0.05) with the introduction of the DT. Dual Task Cost (DTC) demonstrated significant effects for Vision (*p* = 0.008, ηp^2^ = 0.62), with values varying significantly (*p* < 0.05) among different vision conditions, especially in CE and Flash conditions. Visual Dependency Quotient (VDQ) analyses revealed significant effects of condition (*p* < 0.001, ηp^2^ = 0.84), with significant changes observed in CE/OE and Flash/OE conditions (*p* < 0.05). Significant effects were observed for Cognitive performance in the Challenge condition (*p* < 0.001, ηp^2^ = 0.86), with decreased performance with cognitive task challenges, particularly in Flash and Goggles conditions (*p* < 0.05). In conclusion, cognitive tasks, especially challenging ones, and visual variations significantly impact postural balance in adolescents with ID.

## 1 Introduction

Intellectual disability (ID) is a neurodevelopmental disorder impacting approximately 3% of the population, characterized by deficits in intellectual functioning and adaptive behavior, resulting in challenges across various aspects of daily life (1). Among the concerns faced by adolescents with ID, difficulties in postural balance have been documented (2–6), potentially leading to falls (7). These challenges may be attributed to deficits in visual perception, proprioceptive awareness, and vestibular inputs (4).

Given the importance of postural balance in navigating daily activities, it is necessary to understand the mechanisms involved. The postural control system, comprising postural orientation and equilibrium, strives to uphold an appropriate relationship between body parts and stabilize the body’s center of mass during various activities (8). Sensory systems, including visual, vestibular, and somatosensory, play essential roles in postural control and balance (8). Postural control necessitates significant cognitive resources, evident even in quiet standing through increased reaction times when compared to sitting with support. The complexity of postural tasks correlates with heightened cognitive processing demands (9, 10).

Considering adolescents with ID, cognitive load, reflecting the demands on cognitive resources during a task, becomes a crucial consideration. These individuals may experience heightened cognitive load due to their condition, potentially exacerbating their challenges in maintaining postural balance. Indeed, cognitive functions represent a large area of impairment in ID and are often assessed through IQ assessments where the score is below 70 (11). Within this population, deficits typically manifest in attention, memory, and executive functions, posing significant challenges to daily functioning (12). When individuals with ID engage in tasks that require cognitive processing, such as dual-task (DT) paradigms, they may experience an increased cognitive load that exceeds available resources (13). This increased cognitive load can impair their ability to effectively allocate attention and cognitive resources toward maintaining postural balance (14). Previous studies have indicated that individuals with ID often face difficulties when concurrently performing motor tasks, including postural balance, and cognitive activities, introducing an additional layer of complexity to their motor control difficulties (15–18). This simultaneous engagement may impact their proficiency in managing daily activities. Importantly, existing studies have primarily focused on investigating the effects of DT using various tests on postural balance, leaving a gap in the literature concerning investigations into the effect of cognitive load, particularly when intensified during the execution of the same test, on postural balance in adolescents with ID.

Furthermore, acknowledging the significance of vision as a primary sensory input for postural control, this study extends its focus to encompass vision variability that may introduce additional layers of complexity to the motor control challenges faced by adolescents with ID. Previous research has shown that engaging in a cognitive task while standing with CE or delayed visual feedback diminishes postural balance in individuals without ID (19–22). According to Wickens’ Multiple Resource Theory of information processing (23), when individuals perform both a cognitive task and visually control an upright stance, interference occurs. This interference arises from the competition for common central resources between cognitive and visual processing streams (24). Consequently, the effects of cognitive tasks on postural balance differ under visual and non-visual conditions.

The present study addresses an important gap in the literature concerning the impact of cognitive load, particularly when performing the same task, on postural balance in adolescents with ID. While previous research has examined the challenges of maintaining postural balance in individuals with ID and the effects of DT on postural stability, uncertainties persist regarding how variations in cognitive task complexity influence postural balance in this population. Therefore, this study aims to provide knowledge about this relationship, which could inform the development of more effective interventions to enhance postural balance and improve the overall quality of life for individuals with ID. Moreover, our study considers the role of vision variability, incorporating an additional layer of complexity to the postural balance challenges faced by adolescents with ID. With the inclusion of conditions that introduce variability in sensory inputs, such as altered visual feedback or visual distractions, our methodology reflects the real-world difficulties experienced by adolescents with ID in maintaining postural balance. This method enables us to investigate how variations in visual conditions interact with cognitive load to influence postural balance, offering a valuable understanding of the multifaceted nature of postural balance difficulties in this population. Hence, this study aims to examine the impact of cognitive load, especially its escalation during the execution of the same test, while varying vision conditions, on postural balance in adolescents with ID. It is hypothesized that increased cognitive load, especially while performing a more challenging cognitive task will exacerbate the difficulties that adolescents with ID have in maintaining postural balance. Furthermore, the effect of cognitive load on postural balance will vary depending on visual variations introducing an additional layer of complexity to the investigation.

## 2 Methods

### 2.1 Participants

The sample size was determined in advance using G*power software (version 3.1.9.2; Heinrich Heine University Düsseldorf, North Rhine-Westphalia, Germany) (25). Parameters such as effect size (Cohen’s f), alpha, power, correlation among repeated measures, and non-sphericity correction (ε) were set at 0.4, 0.05, 0.80, 0.50, and 1, respectively. The chosen parameters were based on established guidelines for statistical analysis and power calculations, consistent with methodologies employed in previous studies (15, 16, 26). A minimum of 13 participants was calculated to achieve the desired power and minimize the risk of Type II statistical error. To account for potential participant withdrawals, additional individuals were recruited beyond the G*power recommendation.

**Figure 1:**
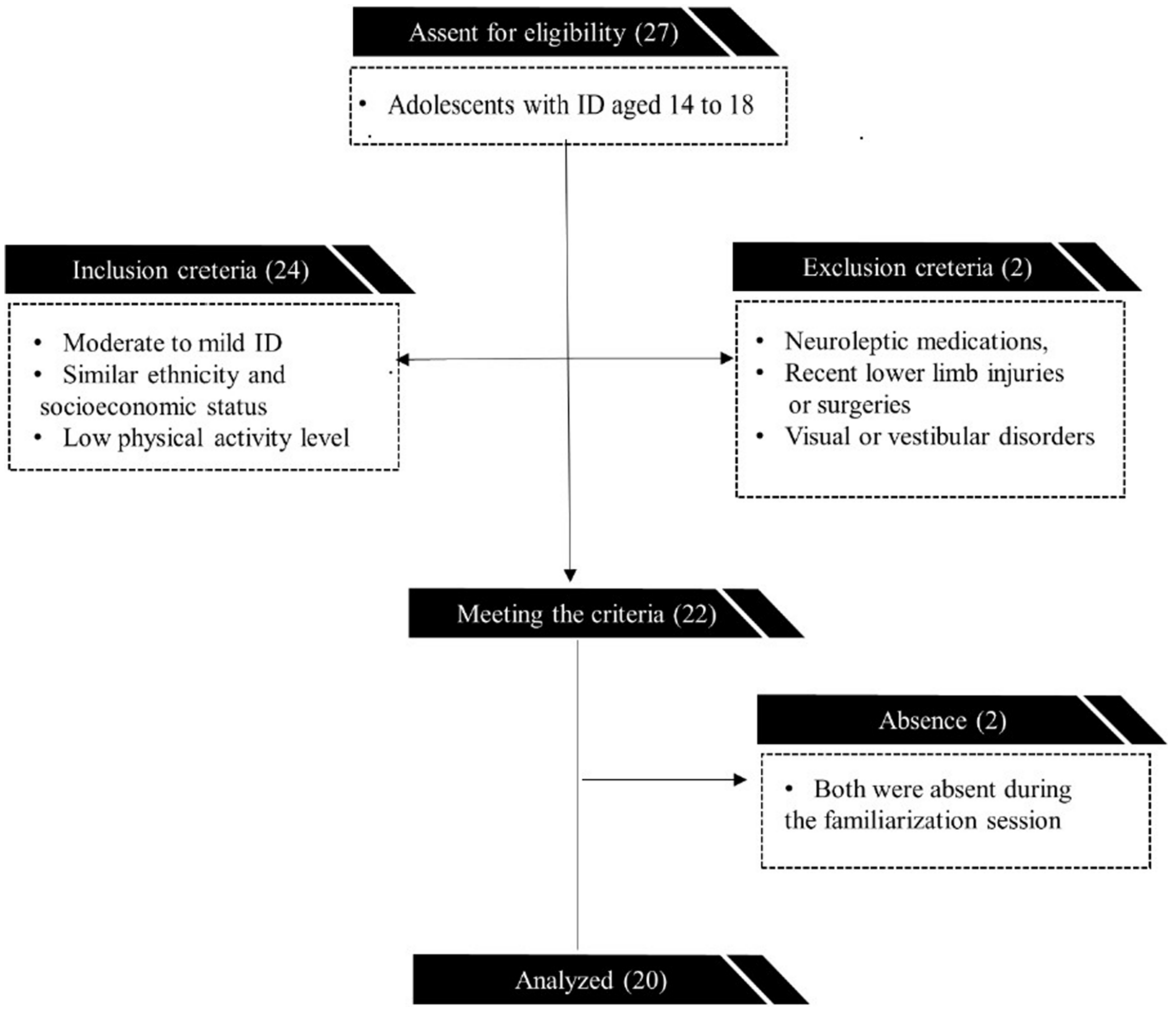
Recruitment process flowchart.

Our recruitment strategy involved a three-stage screening process to define the sample for this study. Initially, we randomly selected 27 adolescents aged 14 to 18 with ID from the special educational center’s database (Figure 1). In the subsequent stage, 22 individuals meeting the specified inclusion and exclusion criteria were identified. The inclusion criteria were as follows: moderate to mild ID, with an average intelligence quotient of 55 ± 3.89, determined by the Wechsler Intelligence Scale for Children fourth edition (27), administered by the center’s psychologist, and similar ethnicity, socioeconomic status, and low physical activity level (International Physical Activity Questionnaire score < 600 Met). Exclusion criteria included the presence of neuroleptic medications, recent lower limb injuries or surgeries, and visual or vestibular disorders. General health status was assessed through medical records provided by the special education center, including information on any existing medical conditions, medication usage, and recent health history. The recruitment criteria were designed to ensure homogeneity within the sample population, thereby minimizing potential confounding variables that could affect the study outcomes. Following the screening process, the final sample size was determined, excluding two individuals who were absent during the familiarization session, resulting in a total of 20 participants (mean age: 16.30 ± 1.17 years, mean height: 167.11 ± 4.35 cm, mean weight: 58.24 ± 5.11 kg).

Before the commencement of the study, a detailed explanation of the experimental protocol was provided to the participants, their parents, and caregivers. Written informed consent was obtained from the parents, and assent was obtained from the participants themselves. This consent process ensured that all participants fully understood the purpose, procedures, and potential risks of the study in a manner appropriate to their developmental level. Moreover, measures were taken to ensure participant privacy and confidentiality by anonymizing all data collected and securely storing any identifiable information. Additionally, participants were assured of their right to withdraw from the study at any time without consequence. The study adhered strictly to the ethical principles set forth in the Declaration of Helsinki and received approval from the ethics committee of Vasile Alexandri University of Bacau, Romania, under approval number 5/2/06.02. 2024.

### 2.2 Study design

This study employed a within-subject, repeated-measures design, comparing postural balance performance within the same individuals across different visual conditions and cognitive task complexities. The study comprised three laboratory visits:

- Initial Visit (First day): This visit was dedicated to familiarization with the experimentation, instructions, and procedures. Participants underwent preliminary assessments to ensure eligibility for the study and the correct execution of the tests.
- Testing Visits (Second and Third days): The subsequent two visits focused on testing sessions. Each testing session occurred on separate days, spaced 1 day apart, to ensure participants were adequately rested between sessions. During these visits, participants completed postural balance assessments under various visual conditions and cognitive task complexities.

All testing sessions were conducted in the morning to minimize variations due to circadian rhythms or daily fatigue.

During these sessions, instructions were provided to participants verbally by a research team member, who has extensive experience in conducting research with individuals with ID. This team member was trained to adapt his communication methods to suit the individual needs and abilities of participants with ID. Besides, the participants were provided simplified explanations, breaking down complex instructions into smaller steps, and using visual aids when appropriate. Participants were encouraged to ask questions and seek clarification if needed, and the team member assessed their understanding through verbal feedback. Furthermore, the assessments took place in the presence of a caregiver familiar with the participants to create a supportive and inclusive environment.

The experiment assessed postural balance, through center of pressure (CoP) recordings, under various visual conditions. These conditions included:

- Open Eyes (OE): Participants maintained a stable bipedal stance with unrestricted vision.
- Closed Eyes (CE): Participants wore a blindfold to eliminate visual input.
- Flash: Participants were briefly exposed to an intense burst of bright light.
- Goggles: Participants wore goggles with prismatic lenses.
- Visual Stimulation (*VS*): Participants were exposed to dynamic color motion videos.

To ensure consistency, all visual conditions were systematically administered, maintaining standardized protocols across participants. Participants were instructed to maintain a stable bipedal stance throughout the assessments. For the OE condition, participants maintained unrestricted vision, while for the CE condition, standardized blindfolds were used to ensure complete occlusion of vision. Goggles were calibrated to induce uniform visual distortion, and the Flash condition was carefully controlled for distance and duration. Additionally, *VS* was standardized in terms of content, duration, and presentation. These measures were implemented to minimize variability in visual input across participants.

Participants engaged in a cognitive task involving verbal fluency (VF) tests during postural balance assessments. The VF task required participants to generate words from specific categories, with variations in task complexity. Postural balance assessments were conducted under Single Task (ST) and Dual Task (DT). In the ST condition, participants solely focused on maintaining postural balance under the visual conditions. In contrast, the DT condition required participants to concurrently perform the cognitive task while maintaining postural balance under the same visual conditions. Throughout the testing sessions, cognitive load and task complexity were systematically manipulated using a cognitive task with and without challenges to elucidate their effects on postural balance. It is important to note that while some participants may have undergone general postural balance assessments or cognitive tests in the past, the specific tasks and conditions investigated in our study were novel to all participants. All assessments were conducted in a standardized manner by trained researchers who were experienced in working with individuals with ID.

### 2.3 Measurements

#### 2.3.1 Postural balance

Participants were instructed to maintain a stable bipedal stance on a static stabilometric platform (PostureWin©, Techno Concept®, Cereste, France) while barefoot. This platform, known for its precision with a sampling frequency of 40 Hz and 12-bit A/D conversion, has been extensively used in postural studies involving individuals with ID (17, 28–32), providing evidence for its validity and suitability. The experiment included five visual conditions: OE, CE, Flash, Goggles, and VS. In the OE condition, participants focused their gaze on a 3 cm target positioned 3 m away, providing a stable visual reference. In the CE condition, participants wore a blindfold to eliminate visual input, allowing investigation into its impact on postural balance. In the Flash condition, participants were briefly exposed to an intense burst of bright light emitted from a standard smartphone flashlight using the “SmartTorch” application, positioned at a distance of 50 cm within a dimly lit environment. This flash of light lasted for 10 s, then the flash automatically stopped. In the Goggles condition, participants wore goggles with prismatic lenses that induced visual distortion. These goggles were made of transparent plastic. The prismatic lenses caused light entering the goggles to bend, resulting in visual distortion and blurriness. In the *VS* condition, participants were exposed to *VS* through dynamic color motion videos on YouTube. Specifically, participants viewed the “Abstract Liquid Background Video (No Sound) — 4 K UHD Abstract Liquid Screensaver.” These videos featured vibrant and fluid patterns in motion, displayed on a computer screen positioned approximately one meter away from the participants. To reduce the influence of sequence effects, the order of the visual conditions was counterbalanced for each participant in our study. Each condition comprised three trials, each lasting 30 s, with a 30-s rest interval to minimize fatigue and learning effects. Participants were allowed to sit during the rest period to maintain postural consistency. The selected parameter was the mean velocity of the Center of Pressure (CoP_Vm_), calculated as the sum of scalar displacements of the CoP divided by the total recording time, expressed in mm/s. CoP_Vm_ reflects the efficiency of the postural control system, with lower values indicating better postural control, making it a reliable measure (33). CoP_Vm_ is commonly used to measure postural stability and has been referenced in several studies across different contexts, including ID (26, 30), Parkinson’s disease (34), multiple sclerosis (35), and functional ankle instability (36). These studies demonstrate its sensitivity in detecting changes in postural stability.

#### 2.3.2 Cognitive task

The VF category test assesses semantic VF by prompting participants to generate words from specific categories like animals, fruits, or colors. Before formal testing, participants completed a practice trial with a different category. Cognitive performance was quantified by the number of correct words produced within the duration of postural balance assessment. Tasks without challenges included generating words for categories like “animals,” “fruits,” and “colors,” while tasks with challenges involved more specific criteria such as “only big animals,” “only small animals,” “animals that swim,” “only small fruits,” or “only fruits that are not yellow.” This test was widely used in individuals with ID (15, 37).

### 2.4 Statistical analyses

The statistical analysis was conducted using SPSS 25.0 (Statistical Package for the Social Sciences Inc., Chicago, IL, United States). The normality of data distribution was confirmed through the Shapiro–Wilk test. To analyze postural balance performance, a two-way ANOVA with repeated measures (5 Vision × 3 Task) was employed to examine the influence of Vision (OE / CE / Flash / Goggles / *VS*) and Task (ST / DT without challenges / DT with challenges) on CoP_Vm_ values. DT costs (DTC) were assessed using the following formula (38): DTC = Condition 1: [(DT without challenges - ST)/ST] * 100 or Condition 2: [(DT with challenges - DT without challenges)/ST] * 100 or Condition 3: [(DT with challenges - ST)/ST] * 100. For the analysis of DTC, a two-way ANOVA with repeated measures (5 Vision × 3 Condition) was conducted to examine the influence of Vision (OE / CE / Flash / Goggles / *VS*) and Condition (Condition 1 / Condition 2 / Condition 3) on DTC values. The Visual Dependency Quotient (VDQ) was calculated using CE/OE, Flash/OE, Goggles/OE, or *VS*/OE. For the VDQ, a two-way ANOVA with repeated measures (4 Condition × 3 Task) was employed to examine the influence of Condition (CE/OE, Flash/OE, Goggles/OE, *VS*/OE) and Task (ST / DT without challenges / DT with challenges) on VDQ values. Concerning cognitive performance, a two-way ANOVA (5 Vision × 2 Challenge condition) was conducted to examine the influence of Vision (OE / CE / Flash / Goggles / *VS*) and Challenge condition (DT without challenges / DT with challenges) on the number of correct answers on the VF task. To assess the practical significance of statistically significant differences, effect sizes for each outcome measure were computed using the partial eta squared (ηp^2^) formula. Effect sizes were categorized as small (0.01 < ηp^2^ < 0.06), moderate (0.06 < ηp^2^ < 0.14), or large (ηp^2^ > 0.14) (39). To account for multiple comparisons, a Bonferroni adjustment was conducted. The level of statistical significance was set at *p* < 0.05.

## 3 Results

The analysis of postural balance performance, the two-way ANOVA, revealed significant main effects for the Vision and Task factors. A significant interaction (Vision × Task) was observed (Table 1).

**Table 1:** ANOVA results.

	*F*	Degree of freedom	*p*	ηp^2^
Postural balance performance				
Vision	64.17	4,16	<0.001	0.91
Task	192.92	2,18	<0.001	0.85
Vision× Dual-task	24.92	8,12	<0.001	0.92
DTC				
Vision	4.45	4,16	=0.008	0.62
Condition	5.77	2,18	=0.010	0.36
Vision × Condition	4.48	8,12	=0.014	0.62
VDQ				
Condition	29.88	3,16	<0.001	0.84
Task	5.62	2,17	=0.013	0.39
Condition × Task	1.78	6,13	=0.179	–
Cognitive performance				
Vision	18.78	4,16	<0.001	0.81
Challenge condition	125.62	1,19	<0.001	0.86
Vision× Challenge condition	2.52	4,16	=0.81	–

DTC, Dual-Task Cost; VDQ, Visual Dependency Quotient.

**Figure 2:**
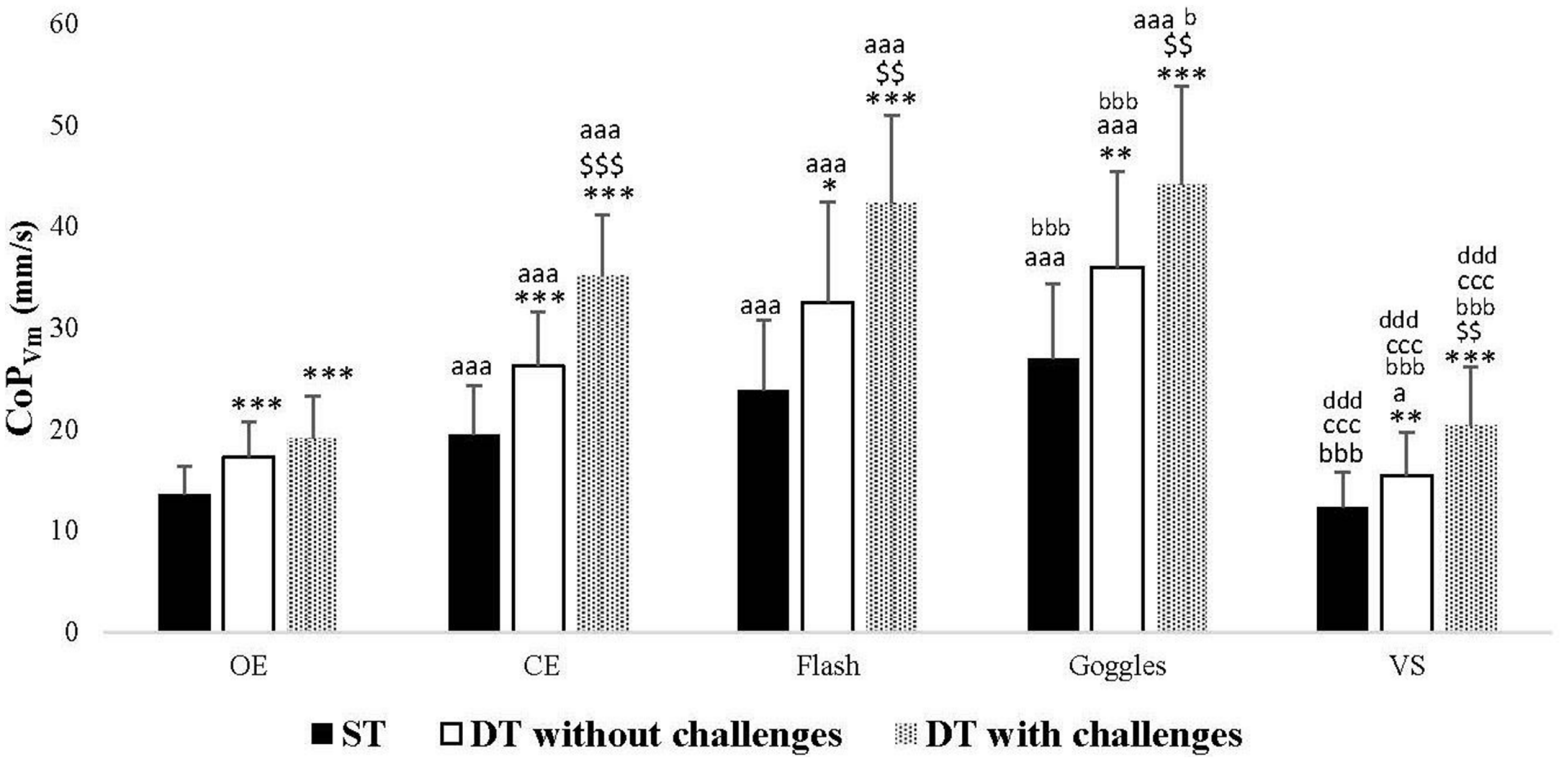
Center of pressure velocity mean (CoP_Vm_) in single task (ST), dual-task (DT) without challenges, and DT with challenges under open eyes (OE), closed eyes (CE), Flash, Goggles, and visual stimulation (*VS*) conditions. ^**^, ^***^: Significance difference (*p* < 0.01, *p* < 0.001) between ST and DT without challenges, and DT with challenges; $$, $$$: Significance difference (*p* < 0.01, *p* < 0.001) between DT without challenges and DT with challenges; aa, aaa: Significance difference (*p* < 0.01, *p* < 0.001) between OE and the rest of the conditions; bbb: Significance difference (*p* < 0.001) between CE and the rest of the conditions; ccc: Significance difference (*p* < 0.001) between Flash and the rest of the conditions; ddd: Significance difference (*p* < 0.001) between Goggles and VS.

Across all tasks (ST, DT without challenges, and DT with challenges), CoP_Vm_ values significantly increased in CE (*p* < 0.001 for all tasks), Flash (*p* < 0.001 for all tasks), and Goggles (p < 0.001 for all tasks) conditions compared to the OE one. There was a significant decrease in CoP_Vm_ values in *VS* condition (*p* = 0.032) compared to the OE one, observed only in the analyses of DT without challenges. Furthermore, CoP_Vm_ values significantly increased in the Goggles condition (*p* < 0.001, p < 0.001, *p* = 0.042, respectively), and significantly decreased in the *VS* condition (*p* < 0.001 for all tasks) compared to the CE one. However, no significant difference was observed between CE and Flash conditions in all tasks. As well, no significant difference was observed between Goggles and Flash conditions in all tasks. Additionally, there was a significant decrease in CoP_Vm_ values in the *VS* condition compared to Goggles (*p* < 0.001 for all tasks) and Flash (p < 0.001 for all tasks) conditions (Figure 2).

Across all visual conditions, *post hoc* analyses revealed an increase in CoP_Vm_ values when introducing DT, both without challenges (OE and Flash: *p* < 0.001, CE: *p* = 0.001, Goggles: *p* = 0.01, *VS*: *p* = 0.003) and with challenges (p < 0.001 for all visual conditions) compared to ST. Furthermore, CoP_Vm_ values exhibited an increase in DT with challenges compared to DT without challenges in all visual conditions: CE (*p* < 0.001), Flash (*p* = 0.001), Goggles (*p* = 0.005), *VS* (*p* = 0.002), with the exception of OE condition (*p* = 0.050) (Figure 2).

Concerning the analysis of DTC, the two-way ANOVA revealed significant main effects for the Vision and Condition factors, along with a significant interaction (Vision × Condition) (Table 1).

**Figure 3:**
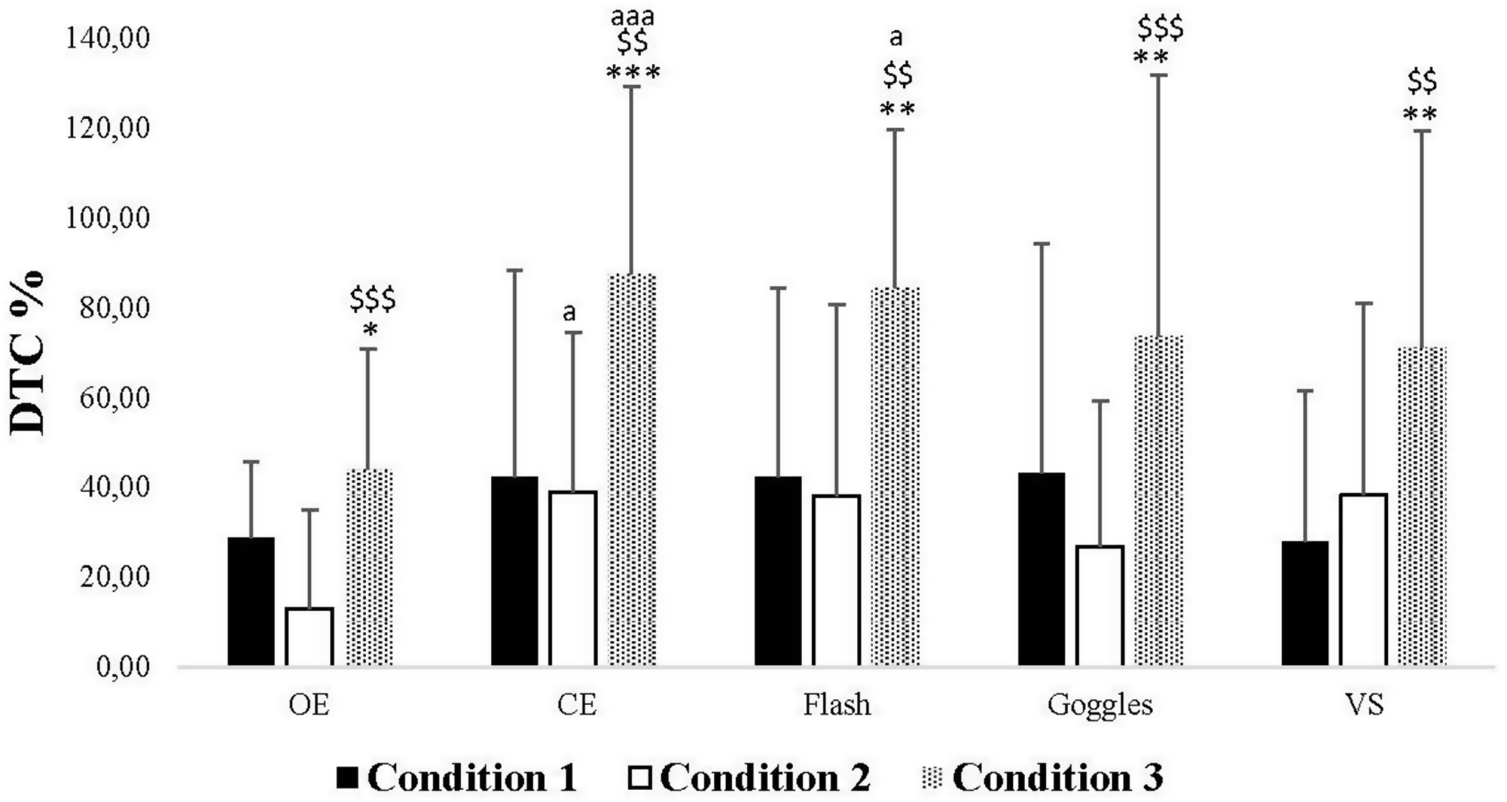
Dual-task costs values (DTC) in Condition 1, 2, and 3 under open eyes (OE), closed eyes (CE), Flash, Goggles, and visual stimulation (*VS*) conditions. ^*^, ^**^, ^***^: Significance difference (*p* < 0.05, *p* < 0.01, *p* < 0.001) between Condition 1 and Condition 2 and Condition 3; $$, $$$: Significance difference (*p* < 0.01, *p* < 0.001) between Condition 2 and Condition 3; aa, aaa: Significance difference (*p* < 0.01, *p* < 0.001) between OE and the rest of the conditions.

In Condition 1, *post hoc* analyses revealed no significant difference in DTC among all vision conditions. In Condition 2, the *post hoc* analyses showed a significant increase in DTC in CE compared to the OE condition (*p* = 0.012). In Condition 3, the *post hoc* analyses indicated a significant increase in DTC in CE and Flash conditions compared to the OE one (*p* < 0.001, *p* = 0.010, respectively). Additionally, the *post hoc* analysis demonstrated no significant difference between Condition 1 and Condition 2. However, there was a significant increase in DTC in Condition 3 compared to Condition 1 in all visual conditions (OE: *p* = 0.046, CE: *p* < 0.001, Flash: *p* = 0.003, Goggles: *p* = 0.003, *VS*: *p* = 0.002), as well as between Condition 3 and Condition 2 (OE: *p* < 0.001, CE: *p* = 0.003, Flash: *p* = 0.003, Goggles: *p* < 0.001, *VS*: *p* = 0.004) (Figure 3).

In terms of the analysis of the VDQ, significant main effects were observed for the Condition and Task factors, although no interaction (Condition × Task) was found (Table 1).

**Figure 4:**
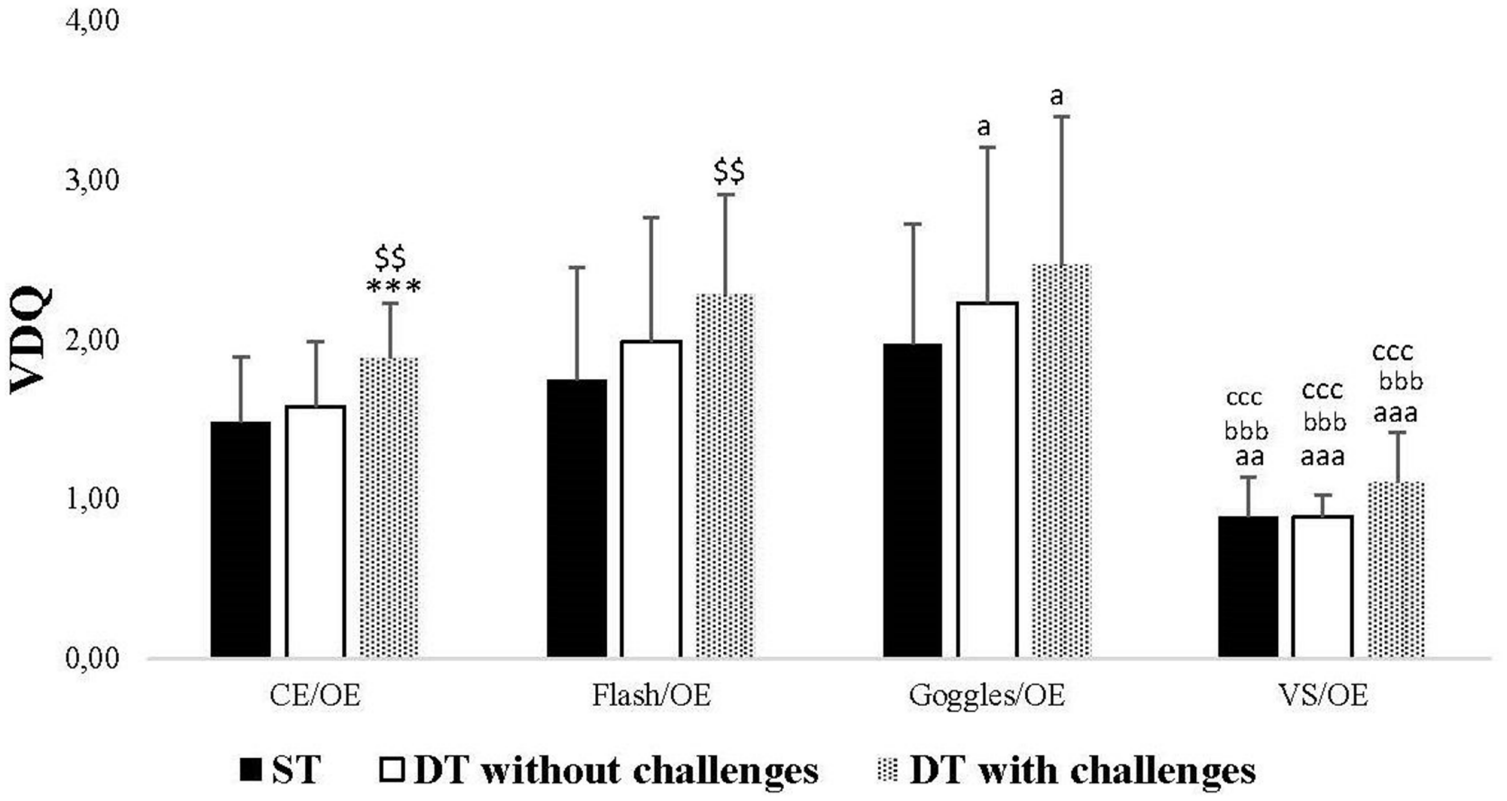
Visual dependency quotient (VDQ) in single task (ST), dual-task (DT) without challenges, and DT with challenges under CE/OE, Flash/OE, Goggles/OE, and visual stimulation (*VS*)/OE conditions. ^**^, ^***^: Significance difference (*p* < 0.01, *p* < 0.001) between ST and DT with challenges; a, aaa: Significance difference (*p* < 0.05, *p* < 0.001) between CE/OE and the rest of conditions; bbb, ccc, ddd: Significance difference (*p* < 0.001) between *VS*/OE and the rest of conditions.

Significant changes in VDQ were noted specifically in CE/OE and Flash/OE conditions. In the CE/OE condition, the VDQ increased significantly in DT with challenges compared to DT without challenges (*p* = 0.002) and ST (*p* < 0.001). Conversely, in the Flash/OE condition, the VDQ increased only in DT with challenges compared to DT without challenges (*p* = 0.009). Additionally, in the ST, there was a significant decrease in VDQ in the *VS*/OE condition compared to CE/OE (*p* = 0.001), Flash/OE (*p* < 0.001), and Goggles/OE (*p* < 0.001) conditions. In the DT without challenges, there was a significant increase in VDQ in the Goggles/OE condition compared to the CE/OE condition (*p* = 0.030), and a significant decrease in VDQ in the *VS*/OE condition compared to CE/OE (*p* < 0.001), Flash/OE (*p* < 0.001), and Goggles/OE (*p* < 0.001) conditions. Similarly, in the DT with challenges, there was a significant increase in VDQ in the Goggles/OE condition compared to the CE/OE condition (*p* = 0.020), and a significant decrease in VDQ in the *VS*/OE condition compared to CE/OE (*p* < 0.001), Flash/OE (*p* < 0.001), and Goggles/OE (*p* < 0.001) conditions (Figure 4).

Regarding the analysis of cognitive performance, significant main effects were found for the Vision and Challenge condition factors, although no interaction (Vision × Challenge condition) was observed (Table 1).

**Figure 5:**
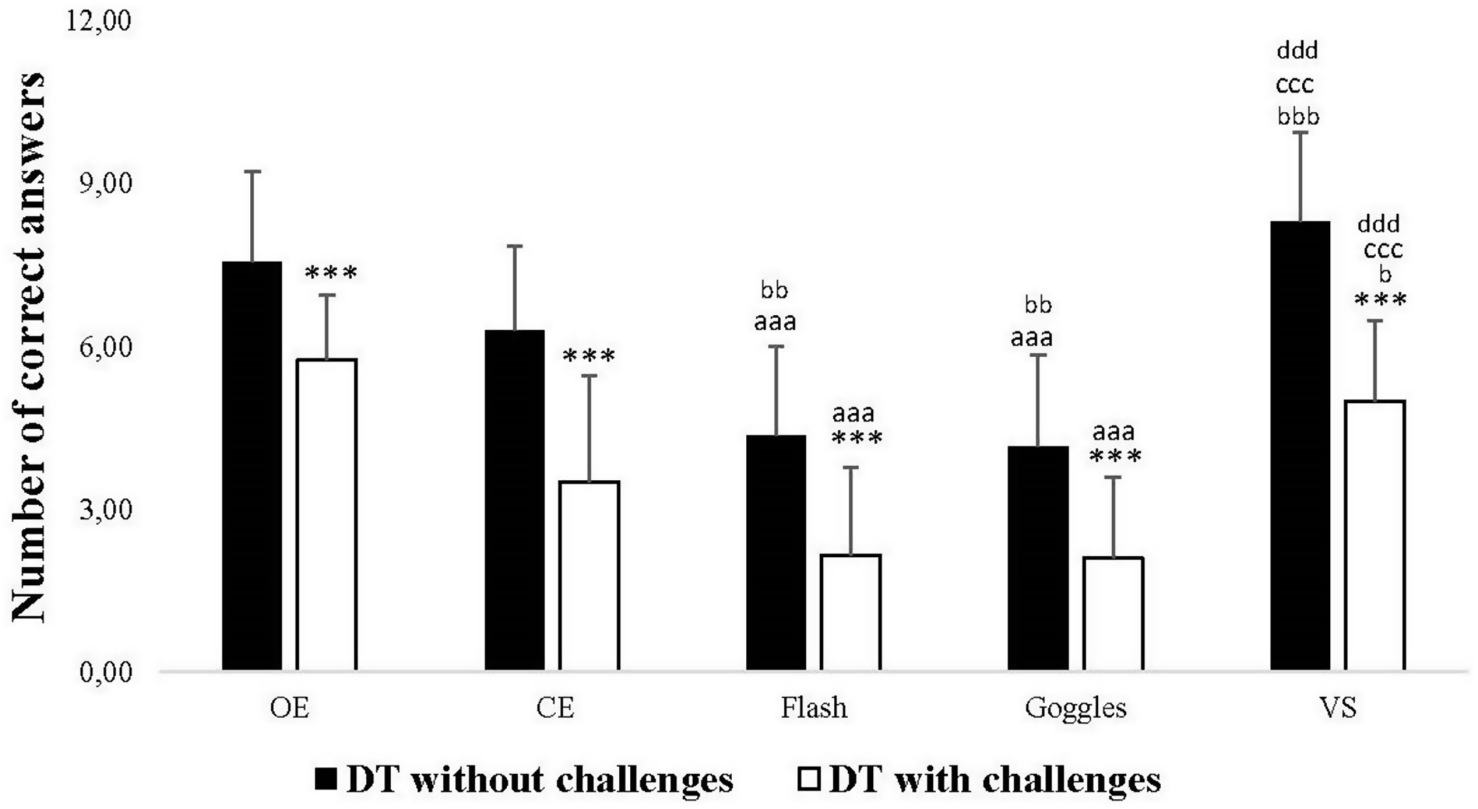
Number of correct answers of the verbal fluency test in dual-task (DT) without challenges and DT with challenges under open eyes (OE), closed eyes (CE), Flash, Goggles, and visual stimulation (*VS*) conditions. ^***^: Significance difference (*p* < 0.001) between DT without challenges and DT with challenges; aaa: Significance difference (*p* < 0.001) between OE and the rest of conditions; bbb: Significance difference (*p* < 0.001) between CE and the rest of conditions; ccc: Significance difference (*p* < 0.001) between Flash and the rest of conditions; ddd: Significance difference (*p* < 0.001) between Goggles and VS.

In DT without challenges, *post hoc* analyses revealed a significant decrease in the number of correct answers in Flash and Goggles conditions (*p* < 0.001 for both) compared to the OE one. However, no significant difference was observed between OE and CE conditions, and between OE and *VS* conditions. There was a significant decrease in Flash (*p* = 0.003), Goggles (*p* = 0.009), and a significant increase in *VS* (*p* < 0.001) compared to CE. No significant difference was observed between Flash and Goggles in the number of correct answers, but there was a significant increase in *VS* compared to both Flash and Goggles conditions (*p* < 0.001, for both). In DT with challenges, *post hoc* analyses revealed a significant decrease in the number of correct answers in Flash and Goggles conditions (*p* < 0.001, for both) compared to the OE one. However, no significant difference was observed between OE and *VS* conditions. There was a significant increase in the number of correct answers in the *VS* condition (*p* = 0.042) compared to the CE one. No significant difference in the number of correct answers was observed between Flash and Goggles conditions, but there was a significant increase in the *VS* condition (*p* < 0.001) compared to both Flash and Goggles conditions. Additionally, the results of *post hoc* analyses indicated a significant decrease in the number of correct answers in DT with challenges compared to without challenges in all visual conditions (*p* < 0.001) (Figure 5).

## 4 Discussion

### 4.1 Effect of visual variability on postural balance

Across all tasks, including ST, DT without challenges, and DT with challenges, the results showed a consistent increase in CoP_Vm_ in conditions of visual deprivation or distortion, such as CE, Flash, and Goggles, compared to OE condition. This finding aligns with the fundamental role of visual input in facilitating spatial orientation and postural adjustments (40). When visual input is available, proprioceptive and vestibular signals align with visual cues to provide accurate feedback on body position and movement, enhancing spatial awareness and balance (41). However, when visual input is compromised or distorted, as observed in conditions like CE, Flash, or Goggles, the central nervous system compensates by placing greater reliance on alternative sensory inputs such as proprioception and vestibular cues to maintain balance (41). In cases where these sensory inputs are additionally compromised, as commonly seen in individuals with ID, it exacerbates postural balance issues due to sensory deficits (4). This compounded effect further disrupts the ability to maintain postural balance when visual input is compromised or distorted compared to normal condition (OE).

Furthermore, the introduction of visual perturbations, such as bright flashes or goggles that distort vision, poses additional challenges to the adaptive mechanisms of the postural control system. Indeed, the bright light from a phone’s flash can cause a brief loss of vision, introducing transient sensory conflicts that challenge the adaptive mechanisms of the postural control system. When exposed to a sudden burst of bright light, the intense luminance can overwhelm the eyes’ visual receptors, resulting in a phenomenon known as flash blindness (42). This brief period of visual impairment disrupts the normal functioning of the visual system, temporarily compromising the brain’s ability to process visual information accurately (42). Compensatory mechanisms may engage, with individuals relying more on proprioception and vestibular cues to offset unreliable visual input. Consequently, the adaptive capabilities of the postural control system may be challenged, potentially leading to transient posture instability (43). Previous research on the effects of ambient lighting on postural sway with OE has suggested reduced efficiency of the visual system under reduced lighting conditions in typically developed individuals (44–46). However, to our knowledge, there is a gap in data regarding the effects of high brightness on postural balance. Similarly, visual distortion caused by wearing goggles introduces a perceptual mismatch between visual and both proprioceptive and vestibular feedback, creating a discrepancy between perceived and actual body position, and reducing postural balance. When individuals wear prismatic goggles, their visual input undergoes distortion, leading to a discrepancy between what their eyes perceive and the body’s internal sense of position, which relies on proprioception and vestibular cues (47). These goggles alter the way the individual sees the world, confounding the brain’s ability to accurately gauge the body’s orientation in space and leading to a state of perceptual confusion. As a result, the integration of sensory inputs necessary for maintaining postural stability is compromised, contributing to increased postural sway. Individuals with ID experience heightened challenges in integrating proprioceptive input without adequate visual cues leading to further decreased postural balance (15, 17, 48). Hence, the reasons why brightness from a phone’s flash or wearing goggles have more pronounced effects than others in this population may stem from the intensity of the perturbation, individual differences in sensory processing, as well as the specific nature of the visual distortion induced by these perturbations. Future studies should clarify the precise mechanisms driving these varying effects in order to understand their implications for the management of postural balance in individuals with ID.

On the other hand, the observed decrease in CoP_Vm_ values in the *VS* condition compared to OE one in the DT without challenges elucidates the potential benefits of *VS* in enhancing postural balance (49), even in individuals with ID, particularly in the absence of additional cognitive challenges.*VS* using color motion, for instance, may positively influence postural balance through its effects on the brain and cognitive performance. Indeed, the use of dynamic visual stimuli, such as color motion, may engage attentional mechanisms and cognitive processing (50). Several studies have discussed the positive effects of colors on the brain (51–53), which may lead to improved postural balance in DT conditions without challenges. Additionally, previous studies have suggested that the effective use of color design in immersive digital contexts can positively influence an individual’s cognitive performance and ID (54). These findings could explain the positive effect of *VS* using color motion on postural balance under DT without challenges. Besides, the psychological impact of visual stimuli should be considered. Colors and visual stimuli have been shown to influence mood, arousal levels (55). Positive affect induced by visually stimulating environments may lead to increased motivation and engagement in postural tasks, potentially enhancing postural balance (56). However, further investigations in future studies are warranted to explore this effect more comprehensively. It is important to note that this effect was not observed in DT with challenges. The lack of a similar effect in the DT with challenging condition suggested that the cognitive demands imposed by the difficult task may overshadow the potential benefits of *VS* on postural balance. When individuals with ID are engaged in more demanding cognitive tasks, the influence of visual stimuli on postural balance may be diminished by the greater attentional and cognitive resources required to perform the task effectively.

### 4.2 Effect of cognitive load on postural balance and cognitive performances

The observed increase in CoP_Vm_ values in DT conditions reflects the competition for cognitive resources between postural balance and cognitive tasks, highlighting the relationship between cognitive and motor functions in individuals with ID. The cognitive-motor interference hypothesis posits that engaging in cognitive tasks concurrently with postural control can lead to impairments in both motor and cognitive performance (15, 57–59). Essentially, this hypothesis highlights the competition for limited cognitive resources within the brain. When individuals perform dual tasks involving both postural balance and cognitive activities, the demands on cognitive resources can interfere with motor performance and vice versa (60, 61). This interference occurs due to the limited capacity of cognitive resources and the brain’s inability to fully allocate these resources to multiple tasks simultaneously. As a result, individuals may experience decreased postural balance and cognitive functioning when engaging in DT activities. Consequently, as individuals with ID engage in cognitive tasks concurrently with postural control, cognitive resources that could have been allocated to maintaining balance are diverted, leading to compromised balance performance (13, 15–18). The increase is exacerbated by the difficulty of cognitive tasks, indicating the intricate interaction between cognitive and motor functions in this population. In individuals with ID, who may already have cognitive deficits or limited cognitive reserve (1), the effects of cognitive-motor interference can be particularly pronounced (15). The introduction of cognitive tasks, especially those requiring increased cognitive load or complexity, may overwhelm the available cognitive resources, leading to disruptions in both motor control and cognitive functioning (15).

Our findings extend previous research by examining the effects of cognitive load within a single test on postural balance in individuals with ID. While previous studies have primarily focused on examining the relationship between cognitive tasks and postural balance in individuals with ID (13, 16–18), few studies have specifically looked at cognitive load. Interestingly, only one study, to our knowledge, has directly compared the effects of different cognitive tests on postural balance in individuals with Down Syndrome (15). This study suggests that adolescents with Down Syndrome experience worsened postural balance when their attention is divided, even when performing simple motor tasks like standing. Additionally, the interference between cognitive tasks and postural balance was more significant during a VF task compared to a working memory task. Therefore, the present study complements the existing literature by highlighting the impact of cognitive load within a single test on postural balance in individuals with ID. The observed decline in cognitive performance during DT conditions with challenges suggested the presence of cognitive challenges reduced cognitive processing efficiency and performance. For instance, a study found that for tasks involving attention demand, response time decreased as time increased, indicating the impact of cognitive load on task performance (62). Additionally, cognitive load has been associated with changes in operational performance, with higher cognitive load leading to decreased performance in complex tasks (63). Therefore, the evidence suggests that a higher cognitive load can lead to reduced cognitive performance, especially in tasks involving attention demand or complex cognitive processing.

Interestingly, the impact of visual perturbations on cognitive task performance varied across different visual conditions. In the DT without challenges condition, the results revealed a significant decrease in the number of correct answers in both the Flash and Goggles conditions compared to the OE one. This decline in cognitive performance suggested that visual perturbations introduced by the Flash and Goggles conditions negatively impacted cognitive task performance, highlighting the role of visual distractions in affecting cognitive processing (64). Interestingly, there were no significant differences observed between OE and CE conditions, or between OE and *VS* conditions. However, significant differences were observed when comparing CE to Flash and Goggles conditions, with Flash and Goggles conditions resulting in a decreased number of correct answers, and *VS* condition showing improved performance compared to CE. These findings suggest that certain visual conditions, particularly those involving perturbations or distortions like Flash and Goggles, may inhibit cognitive task performance, while others such as *VS* may have a neutral or even beneficial effect on cognitive processing. The differential impact of visual conditions on cognitive performance can be attributed to several factors. For example, the nature of visual disturbances, such as sudden flashes of light or distorted vision caused by glasses, might have disproportionately strained cognitive resources, already depleted by their ID, leading to decreased cognitive task performance. Furthermore, psychological effects of visual stimuli, such as their impact on mood and arousal levels, might have played a role in changing cognitive task performance under different visual conditions.

It is important to note that, in our study, we employed the VF category test to assess cognitive performance (15, 37). This test required participants to generate words from specific categories, such as animals, fruits, or colors. Tasks without challenges involved generating words for broad categories like “animals,” “fruits,” and “colors,” requiring relatively simple cognitive processing. Conversely, tasks with challenges imposed more specific criteria, such as “only big animals,” “only small animals,” or “animals that swim,” demanding higher cognitive load and cognitive flexibility. These differences in task complexity likely influenced the observed effects on both postural balance and cognitive performance. Tasks with challenges may have diverted more cognitive resources away from postural control, leading to compromised balance performance. Additionally, individuals with ID may have experienced increased cognitive load when simultaneously performing more challenging cognitive tasks with motor tasks (13), further impacting both postural and cognitive performance.

### 4.3 The interplay between cognitive tasks and vision

The results highlighted the interplay between cognitive task complexity and visual conditions in modulating postural balance in individuals with ID. In Condition 1, where participants performed DT without additional challenges (DT without challenges-ST/ST), the lack of significant differences in DTC between all vision conditions suggested that visual input alone does not substantially affect the allocation of cognitive resources between motor and cognitive tasks. This implies that adolescents with ID are able to effectively manage DT without challenges without experiencing significant interference from varying visual conditions. However, in Condition 2 (DT with challenges - DT without challenges/ DT without challenges), the introduction of cognitive challenges led to a significant increase in DTC in the CE condition compared to the OE one. This suggested that visual deprivation exacerbates conflict for cognitive resources, leading to increased postural balance disturbance, especially as cognitive tasks become more demanding. Indeed, it has been found that engaging in cognitive tasks while standing with CE leads to decreased postural balance (65). Similarly, a previous study investigated how cognitive tasks affect the visual regulation of upright posture and observed that performing cognitive tasks inhibited the visual processing necessary for maintaining postural balance (66). Another study supported the view that interactions between visual processing and cognitive task performance influenced postural balance (19). In Condition 3 (DT with challenges -ST/ST), significant increases in DTC were observed in both CE and Flash conditions compared to the OE condition providing additional light into the relationship between cognitive task complexity and visual conditions. This suggested that the combined effect of cognitive challenges and visual perturbations significantly compromised postural balance. The lack of significant differences between Condition 1 and Condition 2 but the significant increase in DTC in Condition 3 compared to both highlighted the additive effect of cognitive challenges and visual perturbations on postural balance. These results are consistent with the cognitive-motor interference hypothesis, according to which DT places additional demands on cognitive processing, impairing both motor and cognitive performance (15, 59, 67, 68). The observed increases in DTC highlight the strain placed on cognitive resources as individuals strive to maintain balance while simultaneously engaging in cognitive tasks, particularly under conditions of visual deprivation or distortion. Moreover, the examination of VDQ elucidated the degree of reliance on visual feedback for postural balance in individuals with ID. The significant increases in VDQ under visual deprivation and disruption conditions during DT conditions highlighted the increased visual dependence on DT to compensate for cognitive deficits. It appears that individuals with ID rely more on visual cues to alleviate cognitive challenges during postural balance tasks.

This study faces some limitations that should be addressed in future studies. The study sample may not fully represent the diverse population of individuals with ID because participants were recruited in a specific demographic or clinical setting. Therefore, generalization of the results to broader populations of individuals with ID should be done with caution. Additionally, the study focused on individuals with moderate to mild ID, limiting the generalizability of the results to individuals with more severe ID. Moreover, while the study identified associations between visual conditions, cognitive tasks, and postural balance, causal relationships cannot be inferred due to the study’s cross-sectional design. Future studies using experimental or intervention designs could elucidate causal mechanisms underlying these relationships. Another limitation of our study is the potential influence of individual differences in cognitive abilities on the observed effects. While we employed cognitive tasks of varying complexity levels, we did not directly assess participants’ cognitive profiles, such as attention, working memory, or executive functions. These cognitive domains are known to vary widely among individuals with ID and can significantly impact performance on cognitive tasks and motor functions. The lack of individual cognitive assessments limits our ability to elucidate how specific cognitive abilities may have interacted with task demands and influenced postural balance outcomes. Future studies could benefit from incorporating comprehensive assessments of cognitive abilities to better understand their role in shaping the relationship between cognition and motor function in individuals with ID. Importantly, this study did not directly assess certain mechanisms that could explain the observed results. For example, the specific neurophysiological pathways involved in the interaction between visual processing, cognitive load, and postural balance were not investigated. Future studies could use techniques such as electroencephalography to elucidate the neural correlates of cognitive-motor interactions in individuals with ID. Besides, our results may have been influenced by factors such as fatigue, motivation, and participants’ familiarity with postural and cognitive tasks. While we attempted to maintain standardized protocols, fluctuations in fatigue or motivation could have impacted task engagement and postural balance. Also, participants’ prior familiarity with tasks might have influenced their performance, particularly in the cognitive task. However, we acknowledge that these factors were not assessed or addressed, potentially introducing confounding variables. Future studies in this area should incorporate measures to account for these influences.

Despite the limitations, the findings of this study have important practical implications. Given the reliance on visual feedback for postural balance observed in this study, interventions incorporating multisensory training could be beneficial, reducing the reliance on visual stimuli alone. Besides, the findings suggest that integrating cognitive rehabilitation strategies into balance training programs could be particularly beneficial. One effective strategy involves progressively increasing the cognitive difficulty of training exercises to enhance cognitive flexibility and resource allocation during concurrent motor and cognitive tasks. This approach not only improves postural balance but also addresses the cognitive deficits associated with impaired DT performance in individuals with ID. As well, reducing visual distractions and obstacles may help create a safer environment for individuals with ID to practice balance exercises. Providing clear spatial cues can enhance their ability to focus on both motor and cognitive tasks simultaneously. Furthermore, caregivers working with individuals with ID can benefit from understanding the impact of cognitive load and visual perturbations on postural balance and cognitive performance. Implementing strategies to minimize cognitive demands during activities of daily living and academic tasks may help optimize functional outcomes and enhance the overall quality of life. By applying the knowledge gained from this study, practitioners and caregivers can develop more effective interventions that maximize engagement and progress, and ultimately improve the quality of life of individuals with ID in real-world settings.

## 5 Conclusion

In conclusion, our findings demonstrate that visual perturbations and cognitive challenges significantly influence postural balance and cognitive performance in individuals with ID. Future studies should explore additional mechanisms underlying these effects, while practical implications suggest integrating cognitive rehabilitation strategies into postural balance training programs to improve DT performance and the overall quality of life in this population.

## Data availability statement

The data that support the findings of this study are available on request from the corresponding author. The data are not publicly available due to privacy or ethical restrictions.

## Ethics statement

The studies involving humans were approved by the ethics committee of Vasile Alexandri University of Bacau, Romania, under approval number 5/2/06.02. 2024. The studies were conducted in accordance with the local legislation and institutional requirements. Written informed consent for participation in this study was provided by the participants' legal guardians/next of kin.

## Author contributions

GJ: Conceptualization, Data curation, Investigation, Methodology, Project administration, Supervision, Validation, Writing – original draft, Writing – review & editing. DA: Conceptualization, Funding acquisition, Methodology, Project administration, Resources, Supervision, Validation, Writing – review & editing. CM: Funding acquisition, Resources, Software, Validation, Visualization, Writing – review & editing. GR: Methodology, Software, Validation, Visualization, Writing – review & editing. CA: Methodology, Resources, Validation, Visualization, Writing – review & editing. MM: Funding acquisition, Resources, Software, Validation, Visualization, Writing – review & editing. SS: Conceptualization, Formal analysis, Investigation, Methodology, Validation, Writing – review & editing.
